# Fibular allograft and anterior plating for dislocations/fractures of the cervical spine

**DOI:** 10.4103/0019-5413.38587

**Published:** 2008

**Authors:** A Ramnarain, S Govender

**Affiliations:** Department of Orthopedic Surgery, Nelson R Mandela Medical School, University of KwaZulu - Natal, South Africa

**Keywords:** Cervical spine trauma, fresh frozen allograft, fibular allograft, anterior fusion in cervical spine

## Abstract

**Background:**

Subaxial cervical spine dislocations are common and often present with neurological deficit. Posterior spinal fusion has been the gold standard in the past. Pain and neck stiffness are often the presenting features and may be due to failure of fixation and extension of fusion mass. Anterior spinal fusion which is relatively atraumatic is thus favored using autogenous grafts and cages with anterior plate fixation. We evaluated fresh frozen fibular allografts and anterior plate fixation for anterior fusion in cervical trauma.

**Materials and Methods:**

Sixty consecutive patients with single-level dislocations or fracture dislocations of the subaxial cervical spine were recruited in this prospective study following a motor vehicle accident. There were 38 males and 22 females. The mean age at presentation was 34 years (range 19-67 years). The levels involved were C5/6 (*n* = 36), C4/5 (*n* = 15), C6/7 (*n* = 7) and C3/4 (*n* = 2). There were 38 unifacet dislocations with nine posterior element fractures and 22 were bifacet dislocations. Twenty-two patients had neurological deficit. Co-morbidities included hypertension (*n* = 6), non-insulin-dependent diabetes mellitus (*n* = 2) and asthma (*n* = 1). All patients were initially managed on skull traction. Following reduction further imaging included Computerized Tomography and Magnetic Resonance Imaging. Patients underwent anterior surgery (discectomy, fibular allograft and plating). All patients were immobilized in a Philadelphia collar for eight weeks (range 7-12 weeks). Eight patients were lost to follow-up within a year. Follow-up clinical and radiological examinations were performed six-weekly for three months and subsequently at three-monthly intervals for 12 months. Pain was analyzed using the visual analogue scale (VAS). The mean follow-up was 19 months (range 14-39 months).

**Results:**

Eight lost to followup, hence 52 patients were considered for final evaluation. The neurological recovery was 1.1 Frankel grades (range 0-3) and two patients with root involvement recovered. At six months bony trabeculae at the graft-vertebrae interface were noted. There were 12 (20 %) cases of graft collapse and one case of angulation which showed no progression. At six months the VAS was 3 (range 0-6). There was no limitation of neck motion at six months in 47 patients.

**Conclusion:**

Fresh frozen fibular allografts are suitable and cost-effective for anterior fusion in cervical trauma.

## INTRODUCTION

Subaxial cervical spine dislocations are common and represent significant osteoligamentous disruption, instability and neurological deficit.^1^,^2^ A recent review of the literature found closed measures to be successful in achieving reduction in approximately 75-80% of unilateral and bilateral facet dislocations.^1^ Although posterior spinal fusion was the gold standard in the management of subaxial dislocations, the anterior approach has several advantages.^3^,^4^ The use of allografts versus autografts for anterior cervical fusion is controversial. However, successful incorporation of allografts has been reported in trauma,^1^,^5^ degenerative disc disease^6^ and spinal tuberculosis.^7^ We performed a prospective study to assess the incorporation of fresh frozen fibular allografts in instrumented anterior fusion for dislocations of the cervical spine.^8^–^11^

## MATERIALS AND METHODS

Sixty consecutive patients with single-level dislocations or fracture dislocations of the subaxial cervical spine following a motor vehicle collision between 2004 and 2006 were included into the study. There were 38 males and 22 females. The mean age was 34 years (range 19-67 years). The exclusion criteria included polytrauma, head injury (Glascow coma scale <15) and delayed presentation (>48 h).

**Figure 1:**
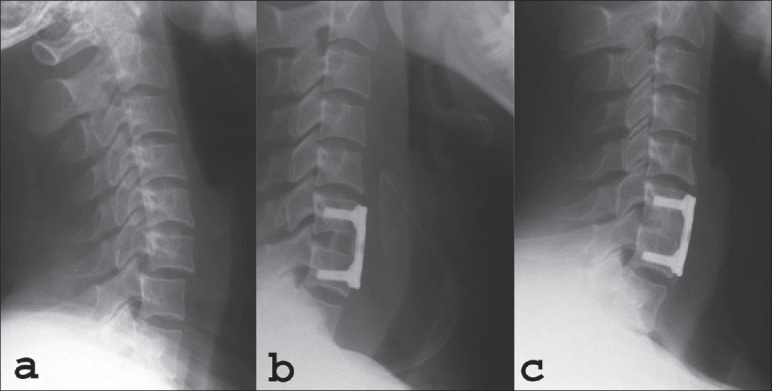
Lateral x-ray (a) cervical spine shows unifacetal dislocation C6/C7. Postoperative lateral x-ray (b) cervical spine of the same patient shows graft and plate in situ. Lateral x-ray (c) cervical spine of the same patient shows graft incorporation with subsidence at six months

The levels involved were C5/6 (*n* = 36), C4/5 (*n* = 15), C6/7 (*n* = 7) [Figure 1a] and C3/4 (*n* = 2). There were 38 unifacet dislocations with nine associated posterior element injuries and 22 were bifacet dislocations. Twenty-two patients had neurological deficit [Table 1]. Co-morbidities included hypertension (*n* = 6), non-insulin-dependent diabetes mellitus (*n* = 2) and asthma (*n* = 1). There were 26 smokers and they smoked an average of 10 cigarettes per day for less than five pack years. All patients were initially managed on skull traction. The patients were positioned supine on a double-split mattress, cone calipers were applied and reduction was effected in the radiology department under conscious sedation and monitoring. Reduction was achieved in 58 patients, the remaining two patients reduced spontaneously following intubation pre surgery. Further imaging included computerized tomography to assess posterior element fractures and magnetic resonance imaging was done to assess for intrinsic cord changes and extrinsic cord compression by a ruptured disc.

**Table 1:** Frankel grading - initial and final presentation

	Frankel grading	
	
	Initial	Final
A	3	2
B	4	2
C	6	4
D	9	5
E	38	47

Patients underwent anterior cervical surgery (discectomy, fibular allograft and plating) between one to two weeks (average seven days post injury). The fibular allografts were harvested and prepared by the national tissue bank (Bone SA) according to the American tissue bank standards of harvesting and storage. A segment measuring 3-5 mm (at a mean cost of $5 per segment) was bevelled to accommodate for the cervical lordosis. The marrow was curetted and both cortices were burred to remove soft tissue remnants. The fibular allograft was then inserted in the prepared disc space and an anterior cervical plate applied. The surgery was performed by the authors and senior residents under supervision. The mean duration of surgery was 75 min (range 64-115 min) and the mean blood loss was 50 ml (range 20-100 ml). There were no wound and neurological complications. All patients were immobilized in a Philadelphia collar for eight weeks (range 7-12 weeks). Bony union was analyzed using the Bridwell criteria by an independent radiologist and orthopedic surgeon. Graft collapse was determined by measuring the graft height initially and at six months. Clinical and radiological examinations were performed six-weekly for three months and subsequently at three-monthly intervals for 12 months. Pain was analyzed using the visual analogue scale (VAS). The neurological status was assessed clinically and documented using the Frankel grading.

## RESULTS

Eight patients were lost to follow-up within a year. In the remaining 52 patients the mean follow-up was 19 months (range 14-39 months).The mean neurological recovery was 1.1 Frankel grades (range 0-3) and two patients with C5 root involvement recovered. The four patients with severe neurological deficit (Frankel A, B) who had no neurological recovery were elderly with co-morbidities.

At three months all patients demonstrated early evidence of graft incorporation which was completed by six months [Figure 1]. There were 12 (22.2%) cases of graft collapse which occurred at level C4/5, C5/6 and C6/7 and one case of angulation which showed no progression. At six months the VAS was 3 (range 0-6). There was no limitation of neck motion at six months in 47 patients and the remaining five patients had restricted motion (flexion and rotation reduced by 25%). There were no cases of disease transmission or wound infections.

## DISCUSSION

Autologous bone has been favored for use in spinal fusion in both the lumbar and cervical spine due to its superior capacity for incorporation and osteoinduction. The incidence of donor site morbidity following iliac crest bone graft harvesting has been cited as ranging from 8-39%.^6^ Donor site pain which represents the most common postoperative symptom often delays mobilization.^12^ Increased operative time and postoperative stay are widely cited as disadvantages of autologous iliac crest bone grafts, especially if graft site complications occur.^12^ Many studies have demonstrated improved fusion rates for allograft bone without the complications of bone graft harvesting.^12^–^15^ The advantage of allograft bone is that it avoids the morbidity associated with donor site complications and is readily available in the desired shape.^16^ The disadvantages of allograft include delayed vascular penetration, slow bone formation, accelerated bone resorption and delayed or incomplete graft incorporation.

Successful fusion of cancellous allografts and dynamic anterior cervical plating were observed in 96% of patients at 12 months.^17^ Fusion was achieved by six months in our study possibly because the segment of fibula used measured 3-5 mm which was smaller than the struts described in the literature.^7^,^17^

Butterman *et al.*,^19^ showed that fresh frozen allografts and freeze-dried allografts had similar fusion rates. Single-level anterior interbody cervical fusion, both fresh-frozen and freeze-dried fibular allografts, incorporated by six months. There were no uniform criteria for radiographic assessment of graft fusion and incorporation.^11^ We used the Bridwell classification of allograft incorporation to assess radiological fusion.

Samartzis *et al.*,^14^ documented a 97,5% fusion rate at a mean of 16 months in patients who had two and three-level anterior cervical fusion using fresh frozen tricortical allograft (35 patients) and tricortical autograft (45 patients).There were no difference in the fusion rate between autografts and allografts. However, Zdeblick and Ducker^15^ noted that patients undergoing anterior cervical fusion had a significantly worse fusion rate using freeze-dried tricortical allograft bone (78% fusion) compared with tricortical autograft bone (92% fusion). Nonunion in one-level procedures was 5% for both autograft and allograft. For two-level procedures, the nonunion rate was 17% for autograft and 63% for allograft. Graft collapse was more commonly seen with freeze-dried allograft (30%) than with autograft (5%).^15^ Freeze-dried allografts are structurally weaker than autografts hence the higher rate of collapse with freeze-dried grafts. In a study of 318 patients who had undergone anterior cervical fusion, Martin *et al.*,^13^ documented a 90% fusion rate with freeze-dried allograft bone in single-level fusions, but this decreased to 72% in two-level fusions possibly due to the longer graft being used in two-level fusion. In addition, the fusion rate with freeze-dried allograft bone was only 50% in smokers compared with 79% in non-smokers.^13^ We had 26 (43%) smokers and two patients with diabetes mellitus. In our study grafts incorporated in all patients with co-morbidities. This may be attributed to single-level fusion and a young patient population when compared to the degenerative group.

Yue *et al.,*^20^ in a long-term follow-up study of 71 patients undergoing anterior cervical decompression and fusion with fibular allograft for degenerative disc disease and fully constrained static plate, reported 93% fusion rate and 82% symptom resolution with a mean follow-up of 7.2 years. They also noted subsidence in 48% cases.^20^ Graft collapse was defined as any loss of graft height and graft subsidence was defined as any migration of the graft into the adjacent vertebral bodies.^20^ The hardware failure and rate of subsidence may indicate that these static plates failed and became ‘dynamic’ before fusion was achieved. Martin *et al.*,^13^ in a study of anterior cervical fusion using fibular allografts reported a 5% incidence of graft subsidence. We had 12 (20 %) cases of graft collapse and no metalware complications. Yue *et al.*,^20^ treated patients with a mean age of 52.7 years requiring surgery for degenerative disc disease while the majority of our patients were young (mean age 34 years), hence our patients possibly had stronger bone resulting in less subsidence. In our study one patient aged 66 years developed a non-progressive segmental kyphosis over the operative level possibly due to “soft” bone.

## CONCLUSION

Fresh frozen fibular allografts are suitable and cost-effective (mean of $5 per patient) for anterior fusion in cervical trauma. There was no increased surgery time and donor site morbidity which is associated with the harvesting of autogenous grafts.
